# Synergistic Role of Aerobic Exercise and Vitamin C in Reducing Hypertension and Restoring Redox–Inflammatory Balance

**DOI:** 10.3390/nu18010153

**Published:** 2026-01-02

**Authors:** Sheraz Ahmad, Khalid Abdul Majeed, Saima Masood, Muhammad Shahbaz Yousaf, Muhammad Bilal Akram, Abdullah Arif Saeed, Habib Rehman

**Affiliations:** 1 Department of Physiology, University of Veterinary and Animal Sciences, Outfall Road, Lahore 54600, Pakistan; drsherazasim@gmail.com (S.A.); drmshahbaz@uvas.edu.pk (M.S.Y.); bilalakram708@gmail.com (M.B.A.); abdullah.arif@uvas.edu.pk (A.A.S.); habibrehman@uvas.edu.pk (H.R.); 2 Department of Anatomy and Histology, University of Veterinary and Animal Sciences, Lahore 54600, Pakistan; saima.masood@uvas.edu.pk

**Keywords:** hypertension, aerobic exercise, vitamin C, antioxidants, gut health, oxidative stress

## Abstract

**Background/Objectives**: Hypertension (HTN) remains a major global concern despite the availability of many antihypertensive medications, each with its own side effects. Lifestyle interventions, such as aerobic exercise and antioxidant-rich foods, represent promising non-pharmacological strategies for hypertension management. This study investigated the combined effects of exercise and vitamin C on anthropometric parameters, blood pressure, gut histology, biochemical markers, hematological profile, inflammatory gene expression, redox status, and stress hormones in L-nitroarginine methyl ester (L-NAME)-induced hypertensive rats. **Methods**: Male Wistar rats (n = 30) were randomly divided into five groups (n = 6/group): control, hypertensive (HTN), hypertensive + exercise (HTN + EX), hypertensive + vitamin C (HTN + VC), and hypertensive + exercise + vitamin C (HTN + EX + VC). Exercise consisted of treadmill training at a low intensity (50 ft/min) for 60 min daily, while vitamin C was administered orally (200 mg/kg/day) for four weeks. Blood pressure, anthropometric parameters, gut histology, inflammatory gene expression, hematological indices, serum biochemistry, oxidative stress markers, and hormonal assays were measured. **Results**: Both exercise and vitamin C individually reduced blood pressure (*p* < 0.05) and increased villi length (*p* < 0.05), upregulated anti-inflammatory cytokine expression in the gut, lowered oxidative stress (assessed through CRP, MDA, and catalase), and reduced stress hormones (cortisol and norepinephrine). The combined intervention (HTN + EX + VC) showed the most pronounced effects, resulting in a greater reduction in blood pressure and reversal of the changes induced by hypertension when compared to the HTN group. **Conclusions**: Exercise and vitamin C were beneficial in lowering blood pressure and improving the adverse changes associated with hypertension.

## 1. Introduction

Significant progress has been made in the management of hypertension treatment and its associated risk factors in the past few decades; however, hypertension continues to be a widespread global issue [1]. Hypertension affects 35% of individuals in developed countries and approximately 40% in developing countries [2]. In Pakistan, about 46% of the population is suffering from hypertension [3]. Hypertension imposes a serious financial burden; for example, in the USA, there is a total cost of 2370 USD per patient [4], and in Pakistan, the mean annual cost for a single hypertensive patient was approximately 321 USD [5]. Beyond financial burden, hypertension is one of the leading causes of major health problems, including stroke, ischemic heart disease, liver dysfunction [6], dyslipidemia [7], imbalanced hematological profile [8], and chronic kidney failure [9]. Additionally, hypertension showed detrimental effects on gut microarchitecture [10]. Although a variety of antihypertensive medications are available, their long-term use is often associated with side effects and financial burden, particularly in low- and middle-income countries, which highlights the need for safe and cost-effective solutions in hypertension management [11]. Lifestyle factors such as physical inactivity, obesity, and unhealthy dietary habits are key modifiable risk factors that play a pivotal role in the onset and progression of hypertension [12]. A sedentary lifestyle has been particularly identified as a key determinant, accounting for nearly 43% of hypertensive cases [13].

Regular physical activity is emerging as one of the most effective non-pharmacological strategies for hypertension [14]. According to the World Health Organization (WHO) guidelines, 2020 recommended that at least 150 min of exercise a week is beneficial for overall health [15]. Exercise has shown significant improvements in vascular compliance, endothelial function [16], angiogenesis [17], and dyslipidemia [18] that contribute to blood pressure regulation.

In addition to physical activity, antioxidant supplementation also emerged as a novel therapy in hypertension management [19]. Among various antioxidants, vitamin C plays a particular role due to its high potential ability to scavenge reactive oxygen species [20], restore nitric oxide bioavailability [21], and improve endothelial dysfunction and blood pressure [22]. The study also indicated that low blood vitamin C levels increased the chances of hypertension [23].

Both exercise and vitamin C improve blood pressure by reducing oxidative stress, improving endothelial function, and vascular tone. Substantial research on both interventions individually is available, but their combined effects on hypertension and related systemic and intestinal alterations remain largely unexplored. We presumed that exercise with vitamin C, in combination, could reduce the BP more efficiently. Therefore, the present study was designed to elucidate the combined effects of exercise and vitamin C supplementation on blood pressure regulation, intestinal microarchitecture, cytokine expression, hematological profile, and health markers in L-nitroarginine methyl ester (L-NAME)-induced hypertensive rats.

## 2. Materials and Methods

### 2.1. Animals

Male Wistar rats (12 weeks old, n = 30) were used in this study. All the experimental protocols were performed with the approval of the Ethical Committee of the University of Veterinary and Animal Sciences (UVAS), Lahore (Directive no. DR/395). The animals were kept in cages with stainless steel-wired mesh tops in a well-ventilated animal house at the Department of Physiology, UVAS. The rats were acclimatized for one week before the start of the experiment. The rats were provided with ad libitum feed and water and kept under a 12-h dark and light cycle with a relative humidity of 60–70%.

### 2.2. Experiment Design

L-nitroarginine methyl ester (L-NAME) (Macklin, Shanghai, China) and vitamin C (Lvyuan Pharmaceuticals, Zhoukou, China) were used in the experiment. Thirty rats (n = 6 per group) were randomly divided into five groups: Negative control (Control), Positive control (HTN), L-NAME + Exercise group (HTN + EX), L-NAME + Vitamin C group (HTN + VC), and L-NAME + Exercise + Vitamin C group (HTN + EX + VC). The rats in the HTN groups received L-NAME dissolved in water (40 mg/kg/day) for 28 days [24]. The rats in the vitamin group were given vitamin C (200 mg/kg/day) by oral gavage for 4 weeks [25].

### 2.3. Exercise Protocol

Rats in the exercise groups underwent a structured low-intensity treadmill training protocol adapted from previously validated rodent endurance-training models [26]. All exercise sessions were performed on a treadmill (SK-21C, Oxygen Fitness, Lahore, Pakistan) under controlled laboratory conditions (room temperature 22 ± 2 °C; relative humidity 50–60%; noise-free environment). During Week 1, rats exercised at 16 ft/min for 30 min per day to allow physiological adaptation. In Weeks 2–4, the workload gradually increased to 50 ft/min for a total of 60 min per day. During Week 1, rats received a 5-min rest following each 15-min exercise interval. From Week 2 onward, rest periods were provided after every 30 min of exercise. This exercise program continued for four weeks, with 7 days/week in the exercise groups [27]. The speed (50 feet/minute) corresponds to approximately 60% of maximal aerobic capacity, thereby remaining within the low-intensity domain [28].

### 2.4. Diet and Anthropometric Parameters

Standard rat chow based on dietary casein was offered during the trial (Table 1).

Body weight (g), body length (cm), abdominal circumference (cm), thoracic circumference (cm), abdominal-to-thoracic circumference ratio, and body mass index (g/cm^2^) were measured at the end of the study. All the measurements were performed as previously described [29,30]. At the end of the experiment, rats were euthanized with thiopental sodium (70 mg/kg i.p.).

### 2.5. Blood Pressure Measurement

Rats were gently placed in a restrainer and given time to acclimate before blood pressure was measured. The blood pressure of the rats was monitored once a week through the tail cuff plethysmography method as per the manufacturer’s protocol (VT200A, Ronseda Electronics Co., limited, Shenzhen, China). The systolic blood pressure (SBP) was measured by taking five readings, and the mean value was used for analysis.

### 2.6. Biochemical Analysis

Blood samples were collected on the last day of the experiment through cardiac puncture, and serum and plasma were separated by centrifugation (3000 rpm; 15 min). The serum was stored at −20 °C until further analysis. At the time of analysis, the serum samples were thawed and concentrations of total protein, albumin, uric acid, total cholesterol (TC), triglycerides (TG), high-density lipoproteins (HDL), and activities of alanine aminotransferase (ALT), aspartate aminotransferase (AST), and alkaline phosphatase (ALP) were measured using commercial kits (Human Diagnostics, Wiesbaden, Germany). The serum low-density lipoproteins (LDL) were calculated using the Friedewald formula [31]:

$$LDL(mg/dL)=TC-HDL-\frac{TG}{5}$$

The atherogenic index was calculated as LDL/HDL [32]. Hemoglobin (Hb), red blood cell (RBC) count, hematocrit (HCT), mean corpuscular volume (MCV), mean corpuscular hemoglobin (MCH), mean corpuscular hemoglobin concentration (MCHC), total white blood cells (WBC), neutrophils, lymphocytes, monocytes, eosinophils, and platelet counts were measured using a hematology analyzer (Mindray, Shenzhen, China). Hematological inflammatory indices such as platelet-to-lymphocyte (PLR), neutrophil-to-lymphocyte ratio (NLR), and systemic immune-inflammation index (SII) were calculated from blood cell counts.

$$PLR=\frac{Platelet\;count}{Lymphococyte\;count}\;\;NLR=\frac{Neutrophil\;count}{Lymphocyte\;count}\;\;SII=\frac{Neutrophil\;count\;x\;Platelet\;count}{Lymphocyte\;count}$$

Serum malondialdehyde (MDA) and catalase levels were measured as previously reported [33]. C-reactive protein (CRP) was measured following the manufacturer’s protocol using a commercial kit (Terasco, Immensee, Switzerland).

### 2.7. Hormonal Assays

The plasma cortisol and norepinephrine were measured using commercial ELISA kits (Bioactive, Vohl, Germany) and (Bio-Techne, Minneapolis, MN, USA), respectively.

### 2.8. Gene Expression

Parts of the small intestine (ileum) were dissected, washed with cold saline, and stored at −20 °C for future analysis. RNA was extracted using TRIZOL (Sigma Aldrich, St. Louis, MO, USA). The RNA in the samples was quantified using a spectrophotometer, and the RNA purity was assessed by measuring the absorbance ratio at 260 and 280 nm (A260/A280). Reverse transcription was performed using the Revert Aid First Strand cDNA Synthesis Kit (Thermoscientific, Waltham, MA, USA). qRT-PCR was completed by using Maxima SYBR Green/ROX (Thermoscientific, Waltham, MA USA) (Table 2). Melting-curve analysis was performed at the end of each qPCR run to verify the specificity of amplification and to rule out primer-dimer formation. PCR amplification of target mRNAs from tissue extracts was performed using a thermocycler. The protocol consisted of 40 cycles, each comprising denaturation at 94 °C for 30 s, annealing for 30 s at 55 °C for TNF-α, 59 °C for IL-10, 60 °C for IL-β, and extension at 72 °C for 1 min. A final extension step was carried out at 72 °C for 10 min after completion of the cycling program. Gene expression levels were normalized to the control sample using the 2^−ΔΔCT^ method, with GAPDH serving as the internal control.

### 2.9. Intestinal Mucosal Microarchitecture

The morphology and collagen deposition of ileum samples were assessed as previously reported [34]. In brief, paraffin-embedded 5 µm sections were stained with hematoxylin and eosin (H&E) and Masson’s trichrome to evaluate ileal mucosal morphometry and fibrosis, respectively. For each sample, 2–3 representative fields were selected from well-preserved areas of the ileum, avoiding folded, torn, or artifact-affected regions. Villus length, crypt depth, and collagen deposition were measured using ImageJ software (NIH, Bethesda, MD, USA, 2012).

### 2.10. Statistical Analysis

Statistical Package for Social Sciences (SPSS Software, Version 20, Chicago, IL, USA) was used for data analysis. Graphs were generated using GraphPad Prism software (Version 8.0; La Jolla, CA, USA). Repeated-measures ANOVA was applied to systolic blood pressure data. One-way ANOVA was applied to the rest of the data, and Tukey’s post hoc test was applied where applicable. Data normality and homogeneity were assessed using the Kolmogorov–Smirnov and Levene’s tests, respectively. *p* < 0.05 was considered significant.

## 3. Results

### 3.1. Anthropometric Parameters

As shown in Table 3, the body weight, abdominal circumference, and thoracic circumference were significantly reduced in the hypertensive (HTN) group compared to the rats in the control group (*p* < 0.05). Exercise (HTN + EX) resulted in significant reductions in body weight and abdominal circumference as compared to the HTN and control groups (*p* < 0.01). In contrast, the rats in the vitamin C group (HTN + VC) showed nonsignificant changes in body weight, abdominal, and thoracic circumferences compared to the HTN group (*p* > 0.05). Notably, the combined intervention (HTN + EX + VC) resulted in the most pronounced reductions in body weight, abdominal circumference, thoracic circumference, as well as body mass index compared to the HTN group (*p* < 0.01).

### 3.2. Blood Pressure

**Figure 1:**
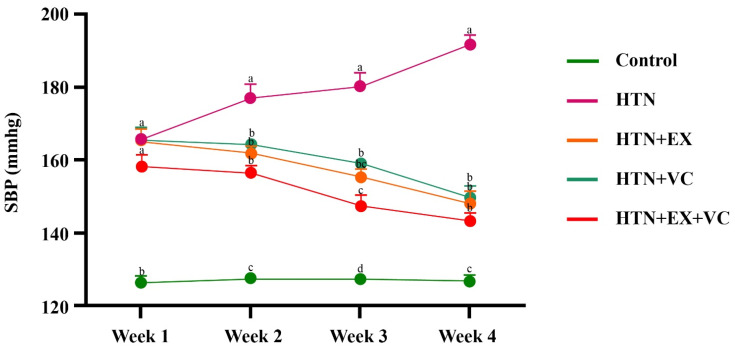
Effects of exercise and vitamin C supplementation on systolic blood pressure (SBP). Control: normal untreated rats; HTN: L-NAME–induced hypertensive rats; HTN + EX: hypertensive rats treated with exercise; HTN + VC: hypertensive rats treated with vitamin C; HTN + EX + VC: hypertensive rats treated with both exercise and vitamin C. Data are presented as mean ± SE (n = 6/group). Lowercase letters a, b, c, and d within the same week indicate significant differences (*p* < 0.05).

Systolic blood pressure (SBP) increased progressively in the HTN group rats compared to the control group throughout the experimental period (Figure 1). Compared to the HTN group, rats in the HTN + EX, HTN + VC, and HTN + EX + VC groups showed a gradual reduction in SBP following the start of their respective treatments.

At the end of week 2, all treatment groups showed significantly reduced (*p* < 0.05) SBP values compared to the untreated hypertensive group (HTN). Rats in the HTN + EX + VC group showed greater reductions in SBP at the end of week 3 compared to the HTN + EX and HTN + VC groups. At the end of week 4, all the intervention groups exhibited significantly lower blood pressure compared to the HTN group (*p* < 0.05), while no significant difference was observed among the intervention groups. However, the control group maintained stable SBP values throughout the 4 weeks, with no significant changes recorded.

### 3.3. Hormonal Assays and CRP

**Figure 2:**
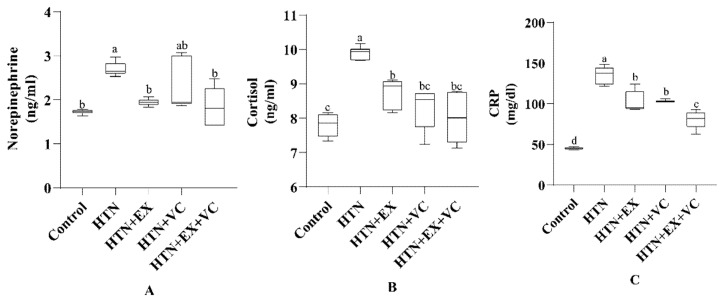
Effects of exercise and vitamin supplementation on (**A**) norepinephrine (**B**) Cortisol (**C**) C-reactive protein. Control: normal untreated rats; HTN: L-NAME–induced hypertensive rats; HTN + EX: hypertensive rats treated with exercise; HTN + VC: hypertensive rats treated with vitamin C; HTN + EX + VC: hypertensive rats treated with both exercise and vitamin C. Data are presented as mean ± SE (n = 6/group). Different superscript letters (a, b, c and d) above the bars indicate statistically significant differences among groups (*p* < 0.05).

Norepinephrine (NE) levels were increased (*p* < 0.05) in the HTN group compared to the control group (Figure 2A). Both exercise groups significantly lowered NE concentrations relative to the HTN group (*p* < 0.05). However, the HTN + VC group showed partial reductions compared to the HTN group (*p* > 0.05). As shown in Figure 2B, cortisol levels were markedly increased in the HTN group compared to the control group (*p* < 0.05). Rats in treatment groups HTN + EX, HTN + VC, and HTN + EX + VC significantly reduced cortisol concentrations relative to the HTN group (*p* < 0.05). However, both the combination group (HTN + EX + VC) and the vitamin C group (HTN + VC) exhibited greater reductions in cortisol levels compared to the HTN group. CRP levels followed a similar trend, showing a significant increase in the HTN group, while the combination treatment group (HTN + EX + VC) exhibited the most pronounced reduction (*p* < 0.01).

### 3.4. Biochemical Analysis

Albumin and total protein levels were significantly increased (*p* < 0.05) in the HTN group compared with the control group (Table 4). Exercise significantly reduces the total protein levels, and this effect was seen in both exercise groups. Cholesterol, triglycerides, and LDL concentrations were significantly increased (*p* < 0.05) in the HTN group compared to the control group, while HDL levels were reduced significantly. Exercise and vitamin C each corrected these abnormalities significantly (*p* < 0.05), but the combination group (HTN + EX + VC) shows the most marked improvement (*p* < 0.01) with lower cholesterol, triglycerides, and LDL and higher HDL concentrations. Consequently, the atherogenic index was also lowest in the combined group. Uric acid levels increased (*p* < 0.05) in the HTN group compared to the control group. Catalase and MDA levels were significantly suppressed in the HTN group. Treatment with exercise or vitamin C in respective groups significantly increased catalase and MDA levels, but the combination group (HTN + EX + VC) showed more pronounced improvements (*p* < 0.01).

### 3.5. Gut Histology and Inflammatory Gene Expression

**Figure 3:**
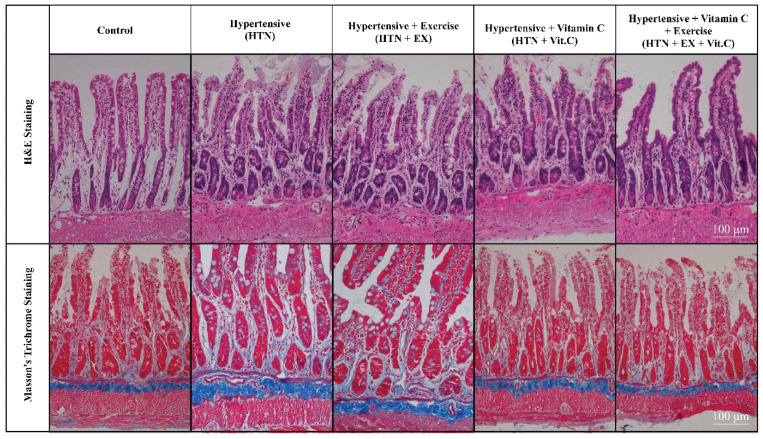
Intestine histology, including villus length and collagen deposition, using H & E staining and Masson’s trichrome Staining. Upper Panel: H & E staining; Lower Panel: Masson’s Trichome staining.

**Figure 4:**
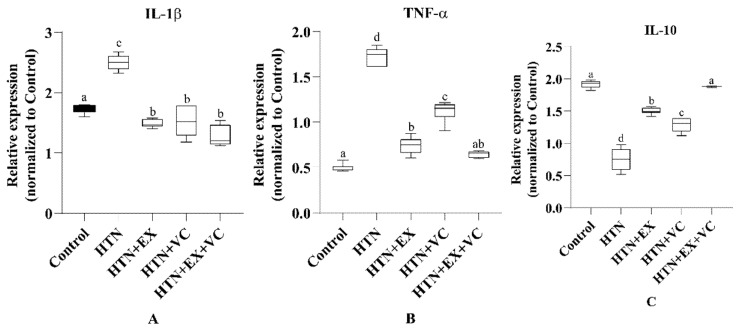
Effects of exercise (EX) and vitamin C (VC) on inflammatory cytokine expression. (**A**) IL-1β, (**B**) TNF-α, and (**C**) IL-10 mRNA expression level. Control: normal untreated rats; HTN: L-NAME–induced hypertensive rats; HTN + EX: hypertensive rats treated with exercise; HTN + VC: hypertensive rats treated with vitamin C; HTN + EX + VC: hypertensive rats treated with both exercise and vitamin C. Data are presented as mean ± SE (n = 6/group). Lowercase letters a, b, c, and d indicate significant differences (*p* < 0.05).

In the HTN group, there was stunted height of villi as well as increased fibrotic area (*p* < 0.05) compared to the control group (Figure 3). The villus length, crypt depth, and collagen deposition of the small intestine improved significantly in the HTN + EX as well as in the HTN + VC groups compared to the HTN group (*p* < 0.05). But the combined treatment (HTN + EX + VC) resulted in better improvements and brought it closer to the control group (*p* < 0.05) (Table 5). Collagen deposition was used to quantify the fibrotic area [34]. Exercise and vitamin C supplementation together significantly improved villus length and reduced fibrotic area in the intestine. There was significant upregulation of TNF-α and IL-1β in the HTN group, while downregulation in the exercise and vitamin C groups (Figure 4). A combination of exercise and vitamin C produced more significant results compared to individual treatments.

### 3.6. Hematological Indices

Hematological analysis showed no significant differences in hemoglobin (Hb), red blood cell (RBC) count, hematocrit (HCT), mean corpuscular volume (MCV), mean corpuscular hemoglobin (MCH), mean corpuscular hemoglobin concentration (MCHC), eosinophils, and platelet count among the groups, as shown in Table 6. In contrast, significant variations were observed in white blood cell (WBC) count, neutrophil, and monocyte count compared to the control group (*p* < 0.05). In the HTN group, there was a significant elevation in total WBC, neutrophil, lymphocyte, and monocyte count compared to the control group (*p* < 0.05). These elevations were significantly attenuated in the HTN + EX, HTN + VC, and HTN + EX + VC groups (*p* < 0.05), except for lymphocyte count. NLR and SII were similar across all the groups (*p* > 0.05). PLR was significantly reduced in the HTN, HTN + EX, HTN + VC, and HTN + EX + VC groups compared to the control group (*p* < 0.05).

## 4. Discussion

The present study demonstrates that combined exercise and vitamin C resulted in the most pronounced antihypertensive activity, which surpasses the individual benefits of both interventions alone in L-NAME-induced hypertension. The combination of exercise and vitamin C not only improved hypertension but also improved anthropometry, small intestinal microarchitecture, inflammatory gene expression, liver and lipid profile, oxidative stress, and stress hormones.

Four weeks of intervention, particularly exercise, significantly lowers body weight and body mass index and reduces the abdominal and thoracic circumferences compared to the control group. These shifts align with previous studies [35,36], which recognize the potential of exercise training to reduce adiposity and central measures associated with obesity, which are independent risk factors for hypertension [37].

In the present study, hypertension deteriorated gut microarchitecture, which is consistent with previous studies [34,38]. The morphological changes are mechanistically linked to an enhanced pro-inflammatory response, which is indicated by downregulation of anti-inflammatory and upregulation of pro-inflammatory cytokines in intestinal tissues of the HTN group. IL-10 is a potent anti-inflammatory cytokine that normally maintains mucosal homeostasis and protects against endothelial dysfunction [39]. The downregulation of IL-10 and upregulation of TNF-α and IL-1β show a sustained inflammatory state, which is consistent with hypertension-driven low-grade inflammation as well as increased production of trimethylamine *N*-oxide (TMAO) in hypertensive models [40,41]. TMAO is linked with upregulation of proinflammatory cytokines like TNF-α and IL-1β, as well as downregulation of IL-10 [42]. Both exercise and vitamin C intake reversed these alterations at the structural and molecular levels. Exercise significantly restored villus length and reduced the fibrotic areas of the intestine. Exercise also has downregulated TNF-α and IL-1β and upregulated IL-10, which demonstrates the well-reported antioxidant and anti-inflammatory effects of exercise [43]. Similarly, vitamin C improved intestinal morphology and downregulated the pro-inflammatory cytokine expression, while upregulating IL-10 expression, which is consistent with the already reported role of vitamin C as a free radical scavenger and cytokine regulator [44].

Hypertensive rats showed elevated WBC counts and neutrophil percentages, which is consistent with previous studies [45,46]. The increase in WBC might be due to hypertension-induced atherosclerosis [47], which leads to activation of the cytokine system. Cytokines like SCF/ck repair endothelial injury, differentiate hematopoietic cells, and lead to myelopoiesis [48]. Exercise reduced leukocytosis by attenuating the low-grade inflammation, as evidenced by low levels of CRP. This effect is primarily mediated through suppression of pro-inflammatory cytokines like TNF-α and IL-1β, along with a decrease in levels of stress hormones like norepinephrine and cortisol [49].

Hypertensive rats have elevated levels of total cholesterol, triglycerides, LDL, and uric acid, along with low levels of HDL, which reflect dyslipidemia and purine metabolism disturbances associated with hypertension as reported in previous studies [50,51]. In the exercise group, total cholesterol and triglycerides were reduced, and this effect is attributed to enhanced lipoprotein lipase (LPL) activity, which hydrolyzes triglycerides into VLDL and chylomicrons and maturation of small HDL particles into larger cholesterol-rich HDL [52]. Exercise also increases ABCA1 expression in macrophages, promoting reverse cholesterol transport, raising HDL levels, thereby reducing atherosclerosis risk [53]. Additionally, exercise regulates nuclear receptors and lipid-related genes, including LXR [54], ApoC3 [55], and PCSK9 [56], all of which improve lipid handling [57]. Aerobic training also upregulates hepatic LDL receptors, enhancing LDL uptake and degradation [58,59]. Together, these mechanisms explain the observed reduction in LDL and rise in HDL, consistent with an earlier study [60]. In the vitamin C group, total cholesterol and triglycerides were also reduced. Vitamin C supports fatty-acid β-oxidation by enhancing carnitine synthesis, which facilitates hepatic lipid metabolism [61]. It also enhances the activity of lecithin/cholesterol acyltransferase (LCAT), an enzyme central to reverse cholesterol transport, enabling cholesterol removal from tissues and excretion via the liver [61]. While vitamin C did not significantly increase HDL, it lowered LDL, likely due to its antioxidant effect, which prevents LDL oxidation and facilitates hepatic clearance [62]. Supporting evidence shows that vitamin C deficiency downregulates hepatic LDL receptors, leading to LDL accumulation [63].

Uric acid levels increased in hypertensive rats, which is consistent with a previous study [64], which may be related to renal impairments associated with hypertension. In the exercise groups, serum uric acid level was reduced, which might be due to improvement in ATP turnover. Exercise reduces adenylate deaminase, due to which precursor synthesis for uric acid production is reduced [65], and it also improves uric acid excretion [66]. Exercise and vitamin C showed the most favorable improvement in the atherogenic index, which shows lipid normalization aligning directly with BP reduction.

Total protein and albumin levels were elevated in hypertensive rats, as aligned with a previous study [50]. This effect results from hemoconcentration caused by reduced plasma volume due to increased vascular tone and enhanced transcapillary fluid loss. Exercise reduces total protein and albumin levels via increased protein oxidation and amino acid metabolism as the body uses amino acids for energy and gluconeogenesis [67].

In the present study, CRP concentrations and MDA levels were markedly elevated in hypertensive rats, reflecting the well-established link between high CRP and hypertension [68]. Hypertension promotes vascular injury and endothelial dysfunction, which stimulate cytokine release and hepatic CRP synthesis, and CRP itself aggravates hypertension by reducing nitric oxide, enhancing oxidative stress, and creating a general inflammatory state in the body [69]. Both exercise and vitamin C lowered CRP and MDA levels in hypertensive rats. The beneficial effects of exercise are largely attributed to its ability to reduce visceral adiposity, a major source of cytokines such as IL-6 and TNF-α, which in turn stimulate hepatic CRP synthesis. Exercise also lowers MDA by mitigating oxidative stress, partly through enhancing antioxidant defenses, as evidenced by the rise in catalase observed in this study [70,71]. Vitamin C improves the antioxidant capacity by enhancing the catalase activity, ultimately lowering lipid peroxidation and MDA levels while also preventing NF-kB activation, thereby suppressing cytokine-driven CRP synthesis [72].

Hypertension raises the levels of norepinephrine (NE) and cortisol, which is consistent with previous studies [73,74]. The elevation in NE is attributed to competitive inhibition of nitric oxide synthase by L-NAME, which reduces NO availability. The absence of NO is believed to increase NE release from sympathetic nerves and adrenal medulla [75]. Exercise reduces NE levels, while vitamin C supplementation resulted in a non-significant change compared to the HTN group. The proposed mechanism behind exercise-induced NE reduction is a rise in prostaglandin E levels, which subsequently reduces NE release from sympathetic nerves [76]. Another proposed mechanism is that exercise reduces ouabain-like digitalis substances, which promote NE reuptake and ultimately lower plasma NE [77]. In our study, low-intensity exercise significantly reduced circulating cortisol levels in hypertensive rats. This finding is consistent with that exercise intensity determines hypothalamic–pituitary–adrenal (HPA) axis activation. High-intensity exercise generally results in cortisol release, while low-intensity exercise causes milder neuroendocrine release and reduced HPA activation. Overall, exercise enhances cortisol clearance and tissue uptake, resulting in lower plasma cortisol levels [78]. Moreover, the beneficial impact of exercise on stress regulation and psychological resilience, as described by Diotaiuti et al. [79], further supports the integrative role of physical activity in maintaining cardiovascular and neuroendocrine balance. Vitamin C also lowers cortisol levels in hypertensive rats. Vitamin C shows this effect by lowering the overall oxidative stress and by interfering with the steroidogenic enzymes involved in cortisol production [80].

## 5. Conclusions

Taken together, exercise and vitamin C provide broad and complementary benefits. Exercise mainly improves blood pressure by lowering stress hormones, improving lipid balance, and reducing general inflammation. Vitamin C strengthens these effects by alleviating oxidative stress, lowering inflammation, protecting the gut lining, and supporting healthier metabolism. When combined, these resulted in improved gut integrity, balanced immune responses, enhanced antioxidant status, and lowered stress hormones. These findings highlight the potential of non-pharmacological strategies, particularly regular exercise with a rich antioxidant diet, for the prevention and management of hypertension.

Among the limitations of this study, the use of an animal model, the relatively small sample size, the short intervention period, and the lack of long-term follow-up should be acknowledged. These factors may limit the generalizability of the findings and highlight the need for larger, longer-duration studies in both animal models and human populations.

## Figures and Tables

**Table 1:** Composition of the experimental diet.

Ingredients	Amount (%)
Casein	14.0
Corn starch	46.6
Dextrin	15.5
Sucrose	10.0
Powdered cellulose	5.0
Soybean oil	4.0
Choline	0.25
L-Cystine	0.18
^1^ Mineral mix	3.5
^2^ Vitamin mix	1.0
Energy components	Kcal (%)
Carbohydrates	73.8
Proteins	13.0
Fats	4.0
Fiber	5.0
Metabolizable energy	3780 Kcal/kg

^1^ Mineral premix provided the following per kg of diet: Calcium, 5000 mg; Phosphorus, 3100 mg; Potassium, 3600 mg; Magnesium, 500 mg; Sodium, 1300 mg; Chlorine, 2000 mg; Iron, 39 mg; Zinc, 35 mg; Manganese, 11 mg; Copper, 6 mg; Iodine, 0.21 mg; Chromium, 1 mg; Selenium, 0.22 mg. ^2^ Vitamin premix provided the following per kg of diet: Vitamin A, 4000 IU; Vitamin D3, 1000 IU; Vitamin E, 7800 IU; Vitamin K, 0.75 mg; Thiamine, 6 mg; Riboflavin, 6.50 mg; Niacin, 30 mg; Pantothenic acid, 16 mg; Folic acid, 2.10 mg; Pyridoxine, 5.80 mg; Biotin, 0.20 mg; B12, 28 µg; Choline, 1250 mg.

**Table 2:** Primers used for target genes.

Target Genes	Forward Primer	Reverse Primer
IL-10	5′-CAATAACTGCACCCACTTCC-3′	5′-ATTCTTCACCTGCTCCACTGC-3′
IL-1β	5′-ACAAGGAGAAGAAAGTAATGA-3′	5′-GCTGTAGAGTGGGCTTAT-3′
TNF-α	5′-GCCCAGACCCTCACACTC-3′	5′-CCACTCCAGCTGCTCCTCT-3′
GAPDH	5′-TGCACCACCAACTGCTTAGC-3′	5′-GGCATGGACTGTGGTCATGAG-3′

IL-10, Interleukin-10; IL-1β, Interleukin-1 beta; TNF-α, Tumor necrosis factor-alpha; GAPDH, Glyceraldehyde-3-phosphate dehydrogenase.

**Table 3:** Effects of exercise and vitamin C on anthropometric parameters (n = 6/group) in hypertensive rats.

Parameter	Control	HTN	HTN + EX	HTN + VC	HTN + EX + VC	*p*-Value
BW (g)	350 ± 5 ^a^	260 ± 6 ^bc^	242 ± 8 ^cd^	278 ± 9 ^b^	217 ± 12 ^d^	<0.001
BL (cm)	14.75 ± 0.40 ^b^	14.83 ± 0.24 ^b^	15.25 ± 0.21 ^ab^	15.33 ± 0.16 ^ab^	16.00 ± 0.44 ^a^	0.010
AC (cm)	16.00 ± 0.31 ^a^	15.16 ± 0.34 ^ab^	14.08 ± 0.23 ^b^	15.66 ± 0.24 ^a^	14.16 ± 0.35 ^b^	<0.001
TC (cm)	15.58 ± 0.35 ^a^	14.25 ± 0.38 ^b^	12.91 ± 0.23 ^c^	15.33 ± 0.10 ^ab^	12.58 ± 0.15 ^c^	<0.001
AC/TC (ratio)	1.02 ± 0.005 ^ab^	1.06 ± 0.006 ^a^	1.02 ± 0.01 ^ab^	1.02 ± 0.01 ^ab^	0.96 ± 0.30 ^b^	<0.001
BMI (g/cm^2^)	1.62 ± 0.94 ^a^	1.18 ± 0.02 ^b^	1.04 ± 0.03 ^bc^	1.18 ± 0.38 ^b^	0.85 ± 0.04 ^c^	<0.001

BW, body weight; BL, body length; AC, abdominal circumference; TC, thoracic circumference; BMI, body mass index. Data are presented as mean ± SE. Lowercase letters a, b, c, and d within the row indicate significant differences (*p* < 0.05).

**Table 4:** Effects of exercise and vitamin C supplementation on serum metabolites and redox status in hypertensive rats.

Parameters	Control	HTN	HTN + EX	HTN + VC	HTN + EX + VC	*p*-Value
Total protein (g/dL)	8.32 ± 0.04 ^c^	9.34 ± 0.08 ^a^	8.42 ± 0.09 ^bc^	9.35 ± 0.11 ^a^	8.69 ± 0.07 ^b^	<0.001
Albumin (g/dL)	3.69 ± 0.05 ^b^	4.20 ± 0.11 ^a^	3.21 ± 0.05 ^c^	4.41 ± 0.1 ^a^	3.58 ± 0.09 ^bc^	<0.001
Uric acid (mg/dL)	3.81 ± 0.07 ^c^	6.46 ± 0.19 ^a^	5.08 ± 0.03 ^b^	6.01 ± 0.20 ^a^	4.03 ± 0.05 ^c^	<0.001
Total cholesterol (mg/dL)	76 ± 3.49 ^c^	126 ± 4.16 ^a^	99 ± 2.97 ^b^	104 ± 1.45 ^b^	84 ± 1.72 ^c^	<0.001
Triglycerides (mg/dL)	39 ± 0.60 ^c^	61 ± 2.36 ^a^	49 ± 1.30 ^b^	54 ± 2.50 ^b^	42 ± 0.67 ^c^	<0.001
HDL (mg/dL)	57 ± 0.72 ^a^	48 ± 2.24 ^c^	55 ± 1.17 ^ab^	49 ±1.15 ^bc^	56 ± 1.57 ^a^	<0.001
LDL (mg/dL)	12 ± 0.42 ^c^	66 ± 4.77 ^a^	35 ± 3.20 ^b^	45 ± 2.02 ^b^	19 ± 2.69 ^c^	<0.001
Atherogenic index	0.20 ± 0.07 ^a^	1.4 ± 0.14 ^a^	0.63 ± 0.07 ^bc^	0.91 ± 0.05 ^b^	0.35 ± 0.05 ^ab^	<0.001
ALT (U/L)	30 ± 0.61	33 ± 0.42	32 ± 0.87	32 ± 0.55	32 ± 0.49	0.100
AST (U/L)	67 ± 0.70	70 ± 1.49	67 ± 0.76	67 ± 0.71	67 ± 0.65	0.100
ALP (U/L)	36 ± 0.56	38 ± 0.32	38 ± 1.11	38 ± 0.34	37 ± 0.61	0.160
Catalase (KU/L)	10 ± 0.77 ^ab^	6 ± 0.89 ^c^	8 ± 0.39 ^bc^	10 ± 0.30 ^ab^	12 ± 0.48 ^a^	<0.001
MDA (µM)	1.22 ± 0.00 ^c^	1.37 ± 0.01 ^a^	1.30 ± 0.00 ^b^	1.19± 0.01 ^c^	1.17 ± 0.02 ^c^	<0.001

Alanine aminotransferase (ALT), Aminotransferase (AST), Alkaline phosphatase (ALP), High-density lipoproteins (HDL), Low-density lipoprotein (LDL), Malondialdehyde (MDA). Control: normal untreated rats; HTN: L-NAME–induced hypertensive rats; HTN + EX: hypertensive rats treated with exercise; HTN + VC: hypertensive rats treated with vitamin C; HTN + EX + VC: hypertensive rats treated with both exercise and vitamin C. Data are presented as mean ± SE (n = 6/group). Lowercase letters a, b, and c indicate significant differences (*p* < 0.05).

**Table 5:** Effects of exercise and vitamin C on intestinal histology (n = 6/group) in hypertensive rats.

Intestine Histology	Control	HTN	HTN +EX	HTN +VC	HTN + VC + EX	*p*-Value
Villus length (µm)	241 ± 2.91 ^a^	202 ± 3.12 ^c^	222 ± 1.47 ^b^	217 ± 0.76 ^b^	248 ± 0.7 ^a^	<0.001
Crypt depth (µm)	120 ± 1.22 ^a^	96 ± 1.72 ^c^	108 ± 3.26 ^b^	109 ± 0.88 ^b^	118 ± 4.32 ^a^	<0.001
Collagen deposition (%)	39 ± 0.42 ^bc^	48 ± 0.76 ^a^	40 ± 0.47 ^b^	37 ± 0.60 ^b^	34 ± 0.18 ^c^	<0.001

Control: normal untreated rats; HTN: L-NAME–induced hypertensive rats; HTN + EX: hypertensive rats treated with exercise; HTN + VC: hypertensive rats treated with vitamin C; HTN + EX + VC: hypertensive rats treated with both exercise and vitamin C. Data are presented as mean ± SE (n = 6/group). Lowercase letters a, b, and c indicate significant differences (*p* < 0.05).

**Table 6:** Effects of exercise and vitamin C on hematological parameters (n = 6/group) in hypertensive rats.

Parameters	Control	HTN	HTN + EX	HTN + VC	HTN + EX + VC	*p*-Value
HB (g/dL)	10.15 ± 0.15	10.03 ± 0.56	9.99 ± 0.39	10.11 ± 0.07	10.28 ± 0.05	0.170
RBC (10^12^/L)	7.03 ± 0.04	7.09 ± 0.04	7.31 ± 0.15	7.15 ± 0.04	7.24 ± 0.11	0.240
HCT (%)	29.67 ± 0.29	28.89 ± 0.37	28.79 ± 0.28	29.61 ± 0.21	29 ± 0.26	0.120
WBC (10^9^/L)	7.39 ± 0.07 ^c^	9.68 ± 0.16 ^a^	9.01 ± 0.05 ^b^	9.25 ± 0.28 ^ab^	8.88 ± 0.05 ^b^	<0.001
MCV (fl)	42.17 ± 0.27	40.75 ± 0.64	39.22 ± 0.88	41.43 ± 0.48	41.68 ± 1.19	0.090
MCH (pg)	14.42 ± 0.13	14.14 ± 0.13	13.69 ± 0.25	14.14 ± 0.13	14.96 ± 0.62	0.110
MCHC (g/dL)	34.20 ± 0.42	34.73 ± 0.34	34.93 ± 0.30	34.16 ± 0.35	35.16 ± 0.28	0.200
Neutrophils (10^9^/L)	4.09 ± 0.57 ^d^	5.70 ± 0.12 ^a^	4.97 ± 0.04 ^bc^	5.31 ± 0.12 ^b^	4.85 ± 0.03 ^c^	<0.001
Lymphocytes (10^9^/L)	2.78 ± 0.03 ^a^	3.53 ± 0.11 ^b^	3.47 ± 0.18 ^b^	3.36 ± 0.09 ^b^	3.40 ± 0.12 ^b^	<0.001
Monocytes (10^9^/L)	0.18 ± 0.009 ^c^	0.30 ± 0.009 ^a^	0.23 ± 0.009 ^b^	0.26 ± 0.007 ^b^	0.23 ± 0.010 ^b^	<0.001
Eosinophils (10^9^/L)	0.16 ± 0.027	0.22 ± 0.008	0.20 ± 0.009	0.20 ± 0.010	0.20 ± 0.007	0.120
Platelet count (10^9^/L)	692 ± 8.68	690 ± 8.10	695 ± 6.08	689 ± 9.50	685 ± 7.47	0.930
NLR	1.46 ± 0.01	1.62 ± 0.06	1.45 ± 0.08	1.58 ± 0.32	1.43 ± 0.12	0.070
PLR	249 ± 5 ^a^	196 ± 7 ^b^	203 ± 110 ^b^	206 ± 6 ^b^	203 ± 9 ^b^	<0.001
SII	1017 ± 6	1120 ± 48	1010 ± 58	1093 ± 30	984 ± 42	0.120

Hemoglobin (Hb), Red blood cell (RBC), HCT (Hematocrit), White blood cell (WBC), Mean Corpuscular Volume (MCV), Mean Hemoglobin Concentration (MCH), Mean Corpuscular Hemoglobin Concentration (MCHC), Neutrophil-to-Lymphocyte Ratio (NLR), Platelet-to-Lymphocyte Ratio (PLR), and Systemic Immune-Inflammation Index (SII). Control: normal untreated rats; HTN: L-NAME–induced hypertensive rats; HTN + EX: hypertensive rats treated with exercise; HTN + VC: hypertensive rats treated with vitamin C; HTN + EX + VC: hypertensive rats treated with both exercise and vitamin C. Data are presented as mean ± SE (n = 6/group). Lowercase letters a, b, c, and d indicate significant differences (*p* < 0.05).

## Data Availability

The datasets generated during and/or analyzed during the current study are available from the corresponding author on reasonable request.

## Abbreviations

ABCA1	ATP-Binding Cassette Transporter A1
ALP	Alkaline Phosphatase
ALT	Alanine Aminotransferase
ANOVA	Analysis of Variance
AST	Aspartate Aminotransferase
ATP	Adenosine Triphosphate
BMI	Body Mass Index
BP	Blood Pressure
CA	Catalase
CRP	C-Reactive Protein
EX	Exercise training
GAPDH	Glyceraldehyde-3-Phosphate Dehydrogenase
HCT	Hematocrit
HDL	High-Density Lipoprotein
HPA	Hypothalamic–Pituitary–Adrenal axis
IL	Interleukin
LCAT	Lecithin–Cholesterol Acyltransferase
LDL	Low-Density Lipoprotein
HTN	L-NAME (Nω-Nitro-L-arginine methyl ester)
LPL	Lipoprotein Lipase
LXR	Liver X Receptor
MCH	Mean Corpuscular Hemoglobin
MCHC	Mean Corpuscular Hemoglobin Concentration
MCV	Mean Corpuscular Volume
MDA	Malondialdehyde
NE	Norepinephrine
NF	Nuclear Factor (e.g., NF-κB)
NIH	National Institutes of Health
NO	Nitric Oxide
PCR	Polymerase Chain Reaction
PCSK9	Proprotein Convertase Subtilisin/Kexin Type 9
RBC	Red Blood Cells
RNA	Ribonucleic Acid
SCF	Stem Cell Factor
SE	Standard Error
SPSS	Statistical Package for the Social Sciences
SYBR	SYBR Green (fluorescent dye for qPCR)
TC	Total Cholesterol
TG	Triglycerides
TMAO	Trimethylamine *N*-oxide
TNF	Tumor Necrosis Factor
